# Neglected acidity pitfall: boric acid-anchoring hole-selective contact for perovskite solar cells

**DOI:** 10.1093/nsr/nwad057

**Published:** 2023-03-03

**Authors:** Huanxin Guo, Cong Liu, Honglong Hu, Shuo Zhang, Xiaoyu Ji, Xiao-Ming Cao, Zhijun Ning, Wei-Hong Zhu, He Tian, Yongzhen Wu

**Affiliations:** Key Laboratory for Advanced Materials and Joint International Research Laboratory of Precision Chemistry and Molecular Engineering, Feringa Nobel Prize Scientist Joint Research Center, Institute of Fine Chemicals, Frontiers Science Center for Materiobiology and Dynamic Chemistry, School of Chemistry and Molecular Engineering, East China University of Science and Technology, Shanghai 200237, China; Key Laboratory for Advanced Materials and Joint International Research Laboratory of Precision Chemistry and Molecular Engineering, Feringa Nobel Prize Scientist Joint Research Center, Institute of Fine Chemicals, Frontiers Science Center for Materiobiology and Dynamic Chemistry, School of Chemistry and Molecular Engineering, East China University of Science and Technology, Shanghai 200237, China; Key Laboratory for Advanced Materials and Joint International Research Laboratory of Precision Chemistry and Molecular Engineering, Feringa Nobel Prize Scientist Joint Research Center, Institute of Fine Chemicals, Frontiers Science Center for Materiobiology and Dynamic Chemistry, School of Chemistry and Molecular Engineering, East China University of Science and Technology, Shanghai 200237, China; Key Laboratory for Advanced Materials and Joint International Research Laboratory of Precision Chemistry and Molecular Engineering, Feringa Nobel Prize Scientist Joint Research Center, Institute of Fine Chemicals, Frontiers Science Center for Materiobiology and Dynamic Chemistry, School of Chemistry and Molecular Engineering, East China University of Science and Technology, Shanghai 200237, China; Key Laboratory for Advanced Materials and Joint International Research Laboratory of Precision Chemistry and Molecular Engineering, Feringa Nobel Prize Scientist Joint Research Center, Institute of Fine Chemicals, Frontiers Science Center for Materiobiology and Dynamic Chemistry, School of Chemistry and Molecular Engineering, East China University of Science and Technology, Shanghai 200237, China; Key Laboratory for Advanced Materials and Joint International Research Laboratory of Precision Chemistry and Molecular Engineering, Feringa Nobel Prize Scientist Joint Research Center, Institute of Fine Chemicals, Frontiers Science Center for Materiobiology and Dynamic Chemistry, School of Chemistry and Molecular Engineering, East China University of Science and Technology, Shanghai 200237, China; School of Physical Science and Technology, ShanghaiTech University, Shanghai 201210, China; Key Laboratory for Advanced Materials and Joint International Research Laboratory of Precision Chemistry and Molecular Engineering, Feringa Nobel Prize Scientist Joint Research Center, Institute of Fine Chemicals, Frontiers Science Center for Materiobiology and Dynamic Chemistry, School of Chemistry and Molecular Engineering, East China University of Science and Technology, Shanghai 200237, China; Key Laboratory for Advanced Materials and Joint International Research Laboratory of Precision Chemistry and Molecular Engineering, Feringa Nobel Prize Scientist Joint Research Center, Institute of Fine Chemicals, Frontiers Science Center for Materiobiology and Dynamic Chemistry, School of Chemistry and Molecular Engineering, East China University of Science and Technology, Shanghai 200237, China; Key Laboratory for Advanced Materials and Joint International Research Laboratory of Precision Chemistry and Molecular Engineering, Feringa Nobel Prize Scientist Joint Research Center, Institute of Fine Chemicals, Frontiers Science Center for Materiobiology and Dynamic Chemistry, School of Chemistry and Molecular Engineering, East China University of Science and Technology, Shanghai 200237, China

**Keywords:** perovskite solar cells, hole-selective contact, boric acid anchor, acid-induced degradation, long-term stability

## Abstract

The spontaneous formation of self-assembly monolayer (SAM) on various substrates represents an effective strategy for interfacial engineering of optoelectronic devices. Hole-selective SAM is becoming popular among high-performance inverted perovskite solar cells (PSCs), but the presence of strong acidic anchors (such as –PO_3_H_2_) in state-of-the-art SAM is detrimental to device stability. Herein, we report for the first time that acidity-weakened boric acid can function as an alternative anchor to construct efficient SAM-based hole-selective contact (HSC) for PSCs. Theoretical calculations reveal that boric acid spontaneously chemisorbs onto indium tin oxide (ITO) surface with oxygen vacancies facilitating the adsorption progress. Spectroscopy and electrical measurements indicate that boric acid anchor significantly mitigates ITO corrosion. The excess boric acid containing molecules improves perovskite deposition and results in a coherent and well-passivated bottom interface, which boosts the fill factor (FF) performance for a variety of perovskite compositions. The optimal boric acid-anchoring HSC (**MTPA-BA**) can achieve power conversion efficiency close to 23% with a high FF of 85.2%. More importantly, the devices show improved stability: 90% of their initial efficiency is retained after 2400 h of storage (ISOS-D-1) or 400 h of operation (ISOS-L-1), which are 5-fold higher than those of phosphonic acid SAM-based devices. Acidity-weakened boric acid SAMs, which are friendly to ITO, exhibits well the great potential to improve the stability of the interface as well as the device.

## INTRODUCTION

The hole-transporting layer (HTL) plays a dominant role in the device performance and long-term stability of inverted perovskite solar cells (PSCs) [1–4]. In addition to guaranteeing efficient hole selection and light transmission, the HTL determines the crystallization quality and film morphology of the perovskite absorber layer to a great extent in this device architecture [5]. Generally, the HTLs are a thin film of organic or inorganic p-type semiconductors that are fabricated on transparent conductive oxide substrates such as indium tin oxide (ITO) [6]. Recently, a new strategy of anchoring-based self-assembly monolayer (SAM) was proposed to construct more efficient hole-selective contact (HSC) for inverted PSCs [7–9]. The SAM-based HSC has unique advantages of minimal parasitic absorption, low material consumption and robust interfacial toughness. Moreover, it confers convenient molecular engineering for fine modulation on the energy levels and surface properties of HTLs at molecular and even atomic levels. The introduction of SAM-based HSC has triggered a revolutionary advance of inverted PSCs as well as their deployment in tandem photovoltaics [10,11]. However, the detailed working mechanism of such anchoring-based SAM is elusive, especially for the adsorption processes and possible chemical interactions at the ITO–perovskite interface driven by the anchoring groups such as phosphonic acid [12,13]. Moreover, given that ITO is sensitive to an acidic environment, the presence of strong acidic anchors is potentially detrimental to the long-term stability of the resulting devices [14–17].

Herein, we demonstrate for the first time that boric acid, a weak acid, can function as an alternative anchor to construct multifunctional SAM-based HSC for realizing highly efficient and stable inverted PSCs. Importantly, the weakened acidity of a boric acid anchor significantly mitigates ITO corrosion. Theoretical calculations reveal a spontaneous exothermic process of boric acid chemisorption and formation of stable B–O–In linkage. The screening of arylamine structures results in an optimal boric acid-anchoring SAM of **MTPA-BA**, endowing excellent hole selectivity, improved perovskite–substrate contact and reduced interfacial defect. These synergistic merits greatly improve perovskite deposition and hole collection, thus resulting in a substantially enhanced fill factor (FF) in inverted PSCs based on several types of perovskite compositions. The optimal **MTPA-BA**-based devices achieve power conversion efficiencies (PCEs) of 22.6%, 21.0% and 19.3% for standard bandgap (1.57 eV) triple-cation, methylammonium (MA)/Br-free and wide-bandgap (1.68 eV) perovskite, respectively, along with high FF values of ≤85.2%, which is much higher than control devices using traditional SAM or polymeric HSC. More importantly, the corrosion-reduced and coherent perovskite–ITO interface conferred by boric acid-anchoring SAM enables excellent stability of PSCs in both shelf life (ISOS-D-1, T90 > 2400 h) and operation (ISOS-L-1, T90 > 400 h), outperforming devices using phosphonic acid SAM. Therefore, the boric acid-anchoring HSC exhibits great potential to improve the stability of ITO, interface as well as device.

## RESULTS AND DISCUSSION

### **Rational** design **of** boric acid-anchoring **HSC**

**Figure 1.**
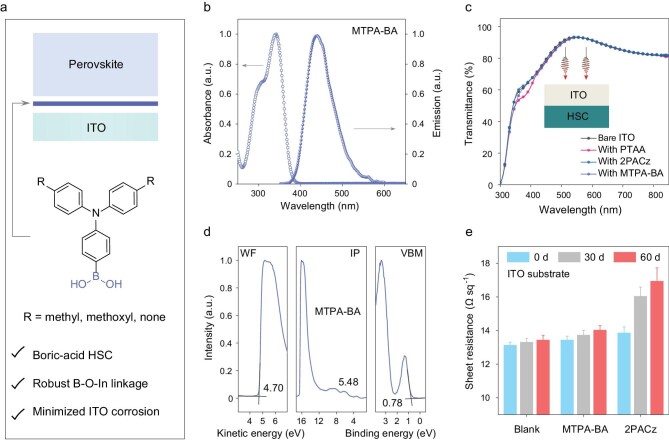
Molecular structure and properties of boric acid-anchoring HSC. (a) Schematic illustration of the molecular structure of boric acid HSC (R is a substituted group, such as methyl/**MTPA-BA**, methoxyl/**MeOTPA-BA**, none/**TPA-BA**). (b) Normalized UV–vis absorption and emission of **MTPA-BA** in dilute solution (dichloromethane, 10^−5^ M). (c) Transmittance spectra of different HSC fabricated on ITO glass; PTAA is the abbreviation for poly[bis(4-phenyl) (2,4,6-trimethylphenyl)amine. (d) UPS results of ITO/**MTPA-BA** sample (IP, ionization potential; VBM, valence band maximum). (e) Variation in sheet resistance of ITO substrate upon soaking in 2PACz and **MTPA-BA** solutions for 48 h and storing in the glove box for 60 days, with data averaged from 10 individual tests, the blank means soaking ITO in only ethanol.

The formation of SAM-based HSC is highly relying on the chemical adsorption that has occurred between the hydroxyl (−OH) group at the ITO surface and acidic anchors, such as phosphonic acid (−PO_3_H_2_), sulfonic acid (−SO_3_H) and carboxylic acid (−COOH) groups in the molecular structure of SAM. According to the Brønsted–Lowry acid–base theory, common *p*–*π* conjugation in these anchors reduces electron density around the oxygen atom in the −OH group and facilitates proton dissociation. The strong acidic groups may corrode the surface structure of ITO and limit the long-term stability of resulting devices [14]. Alternatively, the boric acid group (−B(OH)_2_) is a potential anchor as it can react rapidly with polyhydroxy compounds through a condensation reaction, comparable to a click reaction [18]. More importantly, compared with the aforementioned anchors, the boric acid group does not involve *p*–*π* conjugation and the electronegativity of the boron atom is relatively low, resulting in extremely low Brønsted acidity. Bearing these in mind, a series of boric acid substituted arylamines were designed as potential boric acid SAM (Fig. 1a and Supplementary Fig. 1), including carbazole-based **Cz-BA** and triphenylamine-based **TPA-BA, MTPA-BA, MeOTPA-BA**. Supplementary Fig. 1 shows the density functional theory (DFT) calculations on the distribution and energy levels of their HOMO (highest occupied molecular orbital) and LUMO (lowest unoccupied molecular orbital) [19]. By varying the electron-donating arylamines, we can readily modulate the HOMO level that is highly related to hole extraction. Moreover, the simple molecular structures with a weak intramolecular electronic push–pull effect not only improve photo- and electric-stabilities but also preserve a large HOMO–LUMO gap for optical transparency and electron blocking [20,21].

Figure 1b shows the ultraviolet-visible (UV-vis) absorption and emission spectra of **MTPA-BA** (the best one for PSCs application as shown below) in dilute dichloromethane solution. It exhibits an absorption band at 340 nm and an emission band at 440 nm. The narrow absorption range ensures a high transmittance after coating on ITO substrate (Fig. 1c), which is critical for maximizing the photocurrent of inverted PSCs [12]. The strong emission character makes it become an efficient UV light convertor, which converts UV light in solar irradiation to visible light that is highly favorable for a perovskite absorber [22]. Moreover, bright emission can help us to evaluate the uniformity of HSC on ITO substrate (Supplementary Fig. 2). This is not possible for previous phosphonic acid anchor-based SAM (such as 2-[9*H*-carbazol-9-yl] ethyl phosphonic acid, 2PACz) because they are not emissive. Ultraviolet photoelectron spectroscopy (UPS) was used to evaluate the surface energy levels of **MTPA-BA**-coated ITO (Fig. 1d). Combining the work function (WF, 4.70 eV) and the distance between the Fermi level (*E*_F_) and valence band (VB, 0.78 eV), the HOMO level was calculated to be –5.48 eV versus vacuum. This result is in good agreement with the cyclic voltammetry measurement (Supplementary Fig. 3) and well matched with the valance band edge of normal perovskites. The LUMO level was calculated to be –2.23 eV by adding an optical bandgap (3.25 eV) to the HOMO. The well-aligned HOMO and high LUMO energy levels suggest a high hole selectivity of **MTPA-BA** for application in inverted PSCs. As presented in Supplementary Fig. 4, the thermal decomposition and glass transition temperature of **MTPA-BA** are 243 and 106°C, respectively, which are high enough for device fabrication and application. Moreover, the **MTPA-BA** solution under 12 h of continuous light soaking remains colorless and has similar absorption, indicating decent photostability.

### **Weakened** acidity eliminates corrosion **of ITO with** stable anchoring

The acidity of an organic acid can be varied by their adjacent substituent groups. Generally, the incorporation of electron-withdrawing groups can increase acidity, while electron-donating groups show a much smaller influence on the acidity. For example, the p*K*a of −COOH can be decreased from 4.74 (acetic acid) to –0.30 (trifluoroacetic acid), while triphenylamine-substituted acetic acid exhibits p*K*a of 4.55, comparable to that of acetic acid [23]. Given that the various electron-donating arylamines were incorporated to build boric acid SAM, the impact of hole-transporting moiety on the acidity of boric acid is negligible. To estimate the acidity of anchors on the stability of conductive oxides, ITO was placed in a solution of **MTPA-BA** or 2PACz for 48 h. Then, the sheet resistance was measured and tracked to verify the acid-induced degradation of the ITO substrate. As shown in Fig. 1e, just after soaking, the sheet resistance was increased slightly, probably owing to the formation of SAM on the ITO surface as well as partial ITO corrosion. After storing the soaked ITO in air for 60 days, the sheet resistance soared from 13.86 ± 0.35 to 16.93 ± 0.80 Ω sq^−1^ for 2PACz-treated ones (Fig. 1e), while that of **MTPA-BA**-treated ITO presented a negligible change (from 13.44 ± 0.23 to 14.02 ± 0.27 Ω sq^−1^), comparable to bare ITO placed in air for the same duration. We also measured the indium content in the immersion solution by using inductively coupled plasma mass spectrometry (ICP–MS). As shown in Supplementary Fig. 5, the indium concentration of the 2PACz solution (175 ppb) is one order of magnitude higher than that of **MTPA-BA** (18 ppb). To clarify that the stability issue of ITO really comes from a different acidic group, we further measured the indium content of solutions containing a reference compound of *N*-ethylcarbazole (*N*-EtCz) and 4,4′-dimethyl-triphenylamine (MTPA). The results suggest that the degradation of ITO is indeed caused by the strong acidic anchor, while the backbone structure shows a negligible effect.

**Figure 2.**
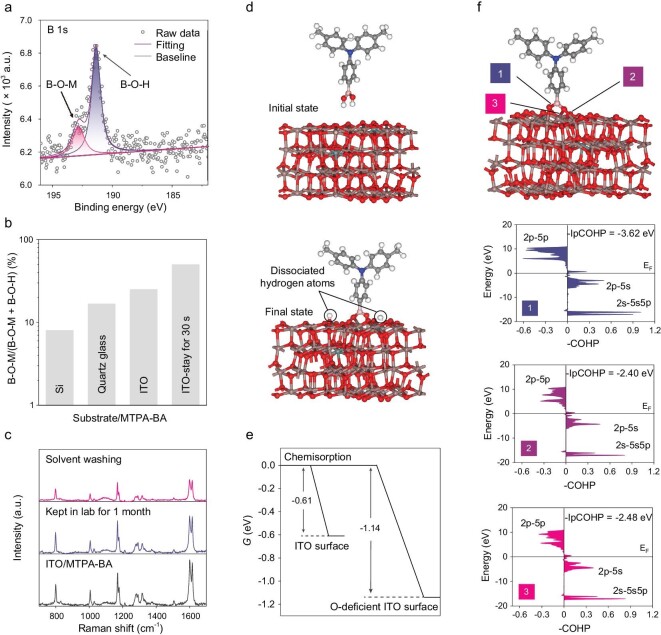
Evaluation of boric acid adsorption on ITO. (a) B 1s XPS result of ITO/**MTPA-BA** sample. (b) B–O–M/(B–O–M + B–O–H) ratio of **MTPA-BA** film deposited on different substrates. (c) Raman spectra of ITO/**MTPA-BA** under different treatment. (d) Initial and final states of dissociative adsorption process of **MTPA-BA** on the ITO surface. The circles mark the dissociated hydrogen atoms. (e) Δ*G* of **MTPA-BA** dissociative chemisorption on the ITO surface (left) and O-deficient ITO surface (right). (f) The COHP analysis of In–O bonding between boric acid and ITO surface (the strength of bond in the tridentate binding state is presented by the −IpCOHP value).

The SAM-base HSC can be prepared by several methods such as immersion and direct spin-coating [24–26]. The simple spin-coating method is favorable because the boric acid-anchoring group can easily react with the ITO surface (as discussed later). X-ray photoelectron spectroscopy (XPS) was employed to verify the boric acid bonding with the ITO. As shown in Fig. 2a, the spin-coated **MTPA-BA** on ITO exhibited B 1s signals at 191.5 and 193.3 eV, corresponding to B–O–H and B–O–M (M means metal ions in ITO substrate), respectively [27,28]. We note that the B 1s XPS results of **MTPA-BA** deposited on silicon wafer and quartz glass were greatly different from ITO, highlighting the contribution of the surface reactive site to the boric acid adsorption (Supplementary Fig. 6). As shown in Fig. 2b, the intensity ratio of B–O–M to the total B 1s signal remarkably increased from surface clean silicon (8.0%) to quartz glass (16.7%) and then ITO substrate (25.0%). These results suggest that the reactive sites at the ITO and quartz glass (SiO_2_) surface are much higher than silicon wafer (Si), which can be attributed to the unique properties of oxides, such as hydroxyl groups and oxygen vacancy [29]. The facile boric acid adsorption was confirmed by placing the **MTPA-BA** solution on ITO before spin-coating. The ratio of the B–O–M signal was doubled from 25.0% to 50.0% (Fig. 2b and Supplementary Fig. 7) within 30 s, indicating quick and efficient SAM formation.

Raman spectroscopy was further used to evaluate the formation and stability of boric acid-anchoring HSC on ITO substrate. As shown in Fig. 2c, the Raman peak at 805 cm^−1^ can be attributed to B–O species [30]. Other peaks at 1006 cm^−1^ (C–N stretch), 1160–1350 cm^−1^ (C–H and C–C stretching vibration in methyl moiety) and 1600 cm^−1^ (C–C and C–N stretches in triphenylamine) confirm the presence of arylamine structures [31,32]. After washing by using deposition solvent or storing the samples in the laboratory environment (air, 20 ± 5°C, 30% humidity) for 30 days, the characteristic Raman peaks were almost unchanged, suggesting a high environmental stability of the boric acid HSC. As exhibited in Supplementary Fig. 8, XPS results show that washed **MTPA-BA** film still retains the unique N 1s and B 1s signals, indicating the stable anchoring of boric acid HSC on ITO substrate. Compared with bare ITO in Supplementary Fig. 9, the In 3d XPS results of the ITO/**MTPA-BA** sample exhibited a negative energy shift, indicative of a strong chemical interaction between the boric acid and the ITO substrate.

### **Theoretical** evaluation **of** boric acid adsorption

Although the concept of SAM-based HSC has been widely accepted in this community, the mechanism for the chemisorption between SAM molecules and the ITO surface is still poorly understood, especially for the unique boric acid anchor. Here the chemical adsorption of boric acid on the ITO surface was studied by using DFT calculation. We chose the (111) plane to build the ITO crystal model to investigate the adsorption as it has been proven to be the most stable surface (Supplementary Fig. 10) [33]. Meanwhile, **MTPA-BA** was used as a model compound to study the thermodynamics of the dissociative adsorption processes (Fig. 2d). As presented in Fig. 2e, the Gibbs free energy change (Δ*G*) of the dissociative adsorption was estimated to be –0.61 eV, suggesting that the adsorption of **MTPA-BA** onto the ITO surface is a spontaneous exothermic process. Crystal occupied Hamilton population (COHP) analysis further verifies the chemical bonding between the boric acid anchor and the ITO surface, reflected by the integration of the projected COHP (IpCOHP) value [34,35]. In order to understand the bonding mechanism, the total interactions were separated into several orbital-pair contributions, including 2s–5s5p, 2p–5s and 2p–5p (2s2p from oxygen atoms of boric acid, 5s5p from indium atoms of ITO). As exhibited in Fig. 2f, the 2s–5s5p and 2p–5s interactions contribute to the formation of bonding states, while the anti-bonding state is stemmed from the 2p–5p orbital-pair contribution. The greatest −IpCOHP value of the O–In bond is estimated to be –3.62 eV, which is higher than several strong acidic anchors (such as −SO_3_H_2_ and −COOH, with −IpCOHP values of –2.92 and –3.28 eV, respectively) and close to the phosphonic acid anchor (−IpCOHP of –3.70 eV) [24]. These results indicated a strong chemical bonding between the boric acid and the ITO surface, and confirmed that boric acid can be used as a promising anchor to establish robust SAM-based HSC for PSCs.

To gain insight into the adsorption of boric acid SAM, we consider the contributions of two types of active sites, i.e. the aforementioned hydroxyl groups and oxygen vacancy at the surface of ITO. Both are capable of establishing a tight bonding between the boric acid group and ITO substrate [36]. DFT calculations suggest that the Δ*G* value for adsorption at the O-deficient ITO surface is more negative (–1.14 eV, Fig. 2e), implying more favorable adsorption via oxygen vacancy sites. To explore the importance of oxygen vacancy in chemisorption, the formation energy of an oxygen vacancy (*E*_Ov_) of several kinds of oxide was further evaluated (Supplementary Figs 10 and 11). As exhibited in Supplementary Fig. 12, the *E*_Ov_ values are 3.12, 2.24, 2.25, 5.79, 4.95 and 3.97 eV for ITO, In_2_O_3_, SnO_2_, Al_2_O_3_, SiO_2_ and TiO_2_, respectively. The lower *E*_Ov_ value indicates easier formation of oxygen vacancy at the surface of oxides. It can be found that the frequently used conductive oxides (i.e. ITO) are featured with low *E*_Ov_ values, further verifying the important roles of oxygen vacancy sites during the chemisorption of SAM-based HSC. These findings convey ignored information that the anchors are preferentially adsorbed at the O-deficient ITO surface. Precise and rapid compensation for the vacancy defects in the oxides surface with anchoring-featured SAM might improve the stability of ITO-involved optoelectronic devices by inhibiting interfacial redox reactions.

### **Boric** acid-anchoring **HSC** enables high**-**quality perovskite deposition

**Figure 3.**
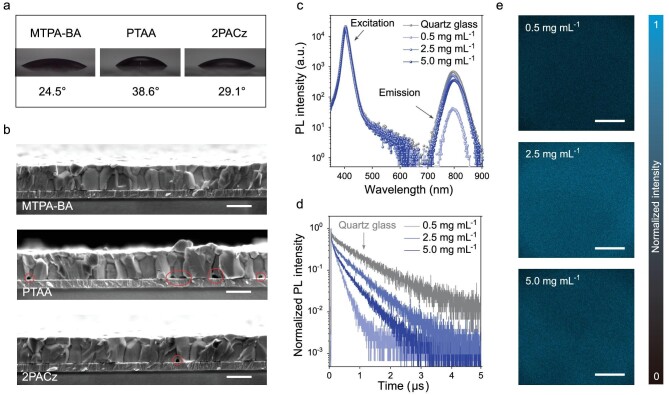
Interface between perovskite and HSC. (a) Contact angles of perovskite inks on three different HSC. (b) Cross-sectional SEM image of ITO/HSC/perovskite (FAMACs-Br_0.05_; the scale bar is 500 nm). (c)–(e) PL emission (405-nm excitation), TRPL (510-nm excitation) and CLSM imaging (514-nm excitation; the scale bar is 200 μm) of perovskite film prepared on ITO/**MTPA-BA**.

The deposition of perovskite film on boric acid-anchoring HSC was investigated, with traditional PTAA and 2PACz as the HSC references. As mentioned above, the selection of HSC can significantly affect the crystallization and quality of the perovskite film [37]. Compared with PTAA and 2PACz, the **MTPA-BA**-coated ITO substrate showed enhanced affinity for perovskite precursor solutions, which were revealed by the quick solution spreading (Fig. 3a and Supplementary Fig. 13). This is crucial to achieve an intimate perovskite–substrate contact. Taking a standard triple-cation perovskite Cs_0.05_(FA_0.95_MA_0.05_)_0.95_Pb(I_0.95_Br_0.05_)_3_ (denoted as FAMACs-Br_0.05_, where FA is formamidinium and Cs is cesium) as an example, we fabricated perovskite films on different HSC via an anti-solvent method and examined the bottom interface by using cross-sectional scanning electron microscopy (SEM) measurement. In Fig. 3b, we observed some voids in the PTAA- and 2PACz-based samples, while the **MTPA-BA** resulted in void-free contact between the perovskite and the substrate. Such an intimate contact at the perovskite–substrate interface is critical for minimizing transport and recombination losses in operational devices.

We found that the excess (unabsorbed) boric acid molecules on the ITO substrate played an important role in improving the electronic quality of the deposited perovskites. Enhanced PL quantum yield (PLQY, Fig. 3c and Supplementary Fig. 14) and time-resolved PL (TRPL, Fig. 3d) of perovskite films were observed by gradually increasing the solution concentration of **MTPA-BA** from 0.5 to 2.5 mg mL^−1^, even though their morphology and crystallinity were nearly identical (Supplementary Figs 15 and 16). The confocal laser scanning microscope (CLSM) mapping of perovskite films (Fig. 3e) further confirmed the beneficial effect of excess boric acid on the PL intensity of perovskite films. Further increasing the concentration to 5.0 mg mL^−1^ resulted in an adverse effect, probably owing to the aggregation of **MTPA-BA**. These results suggested that a certain amount of free boric acid on top of HSC can passivate electronic traps at either the bottom interface or the bulk of the perovskite film.

### **Boric** acid **HSC** achieves efficient **PSCs with** universal **FF** improvement

**Figure 4.**
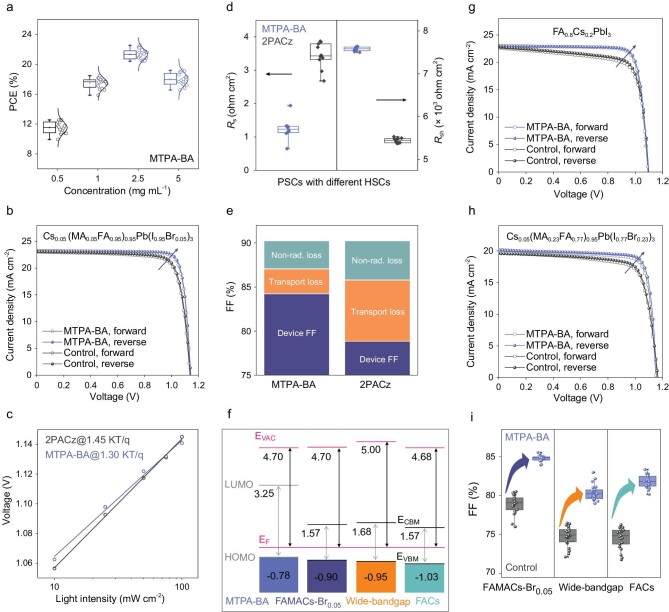
Photovoltaic performance. (a) PCE distributions of PSCs based on **MTPA-BA** with different concentration (FAMACs-Br_0.05_ perovskite). (b) *J–V* curves for FAMACs-Br_0.05_-based devices with different HSC. (c) The plot of the light intensity-dependent *V*_OC_ of the **MTPA-BA**- and 2PACz-based device. (d) Distribution of *R*_s_ and *R*_sh_ from different HSC-based devices (data collected from 10 individual devices). (e) The radiative FF limit of the device consists of non-radiative and transport loss. (f) Energy level comparison of **MTPA-BA** and three perovskites (the data are collected from the literature) [11,13,43]. (g) and (h) Forward and reverse scan *J–V* curves for FACs, wide-bandgap perovskite-based devices, showing an increased FF in **MTPA-BA-**based devices. (i) FF distribution of different PSCs based on **MTPA-BA** and control HSC.

To examine the effectiveness of boric acid-anchoring SAM as HSC, inverted PSCs with a planar structure of ITO/HSC/perovskite/PCBM/C_60_/BCP/Ag (Supplementary Fig. 17) were manufactured, where PCBM is [6,6]-phenyl-C_61_-butyric acid methyl ester and BCP is bathocuproine. The performance of boric acid HSC was first evaluated by using a standard triple-cation FAMACs-Br_0.05_ as the absorber. Supplementary Fig. 18 shows that the triphenylamine-based ones are superior to the carbazole-based **Cz-BA** in this series, and the **MTPA-BA** represents the optimal one as it achieved high short-circuit current density (*J*_SC_), open-circuit voltage (*V*_OC_) and FF synchronously. Therefore, **MTPA-BA** was mainly used in the following device investigation. As exhibited in Fig. 4a, the photovoltaic performance was highly related to the concentration of **MTPA-BA** in the deposition solution. The PCEs was substantially improved from ∼12% (0.5 mg mL^−1^) to >22% (2.5 mg mL^−1^), and further increasing the solution concentration resulted in a decline in the PCE (∼19% for 5.0 mg mL^−1^). These results suggested that the amount of boric acid molecules on ITO was crucial to device performance, consistently with the previous PL characterization. To highlight the importance of the boric acid anchors, anchor-free MTPA was used as HSC to fabricate control devices, which only attained a low PCE of 13%, close to HSC-free devices (Supplementary Fig. 19). This can be ascribed to the dissolution of MTPA by the perovskite precursor solution, resulting in a poor HSC in complete devices. Figure 4b presents the current density–voltage (*J–V*) curves of champion devices based on 2PACz and **MTPA-BA**. The 2PACz-based PSC exhibited a PCE of 21.05% (FF of 80.1%) that was comparable to literature results [12]. Encouragingly, the champion device-based **MTPA-BA** achieved a much higher PCE of 22.62% and FF of ≤85.2%. This is one of the highest FF values amongst inverted PSCs, suggesting the advantages of boric acid HSC for enhancing interfacial electronic quality.

Graphically, the FF is an evaluation of the ‘squareness’ of the *J–V* curve. The FF value of PSCs is mainly limited by the interlayer and the transport losses [38]. We measured the *V*_OC_ as a function of the light intensity from 0.1 to 1 sun (Fig. 4c). The fitted *n* values are 1.45 and 1.30 for 2PACz- and **MTPA-BA**-based PSCs, close to those of high-performance inverted PSCs in the literature [11]. The slight difference in the *n* value should come from the variation in the interfacial energy offset as well as non-radiation recombination at the bottom interface. Supplementary Fig. 20 exhibits *J–V* curves measured under dark conditions, in which the **MTPA-BA**-based device showed a lower leakage current than the 2PACz counterpart. Figure 4d compares the shunt resistance (*R*_sh_) and series resistance (*R*_s_) of **MTPA-BA**- and 2PACz-based devices. By employing empirical formula summarized by Green and Grätzel (Supplementary Equations (3–5)) [39,40], the upper limit of FF (FF_max_, without considering transport loss) of **MTPA-BA**-based devices was calculated to be 87.0%, which exceeds that for the 2PACz-based counterpart (Fig. 4e). When further taking transport loss into account, the calculated FF values of **MTPA-BA** and 2PACz are 84.2% and 78.8%, respectively, consistently with the measured values (errors <2%). These results illustrate that the high-quality and void-free perovskite film assisted by boric acid HSC achieved synergistic optimization of *R*_s_ and *R*_sh_ in the device (Supplementary Table 1), which reduces transport losses and improves FF performance remarkably.

Furthermore, thermally stable MA/Br-free perovskite FA_0.8_Cs_0.2_PbI_3_ (1.57 eV, denoted as FACs) and wide-bandgap perovskite Cs_0.05_(FA_0.77_MA_0.23_)_0.95_Pb(I_0.77_Br_0.23_)_3_ (1.68 eV) were employed to evaluate the universality of boric acid-anchoring HSC in improving photovoltaic performance. Here, we select PTAA and 2PACz as HSC to fabricate reference FACs and wide-bandgap PSC devices, respectively, because they have been proven to achieve decent device performance for these perovskite compositions [41,42]. Figure 4f compared the energy levels of **MTPA-BA** and the three type perovskites (versus *E*_F_), showing a matched band alignment for efficient hole selection. Figure 4g and h shows the champion *J–V* curves of FACs and wide-bandgap perovskite-based PSCs, respectively. Impressively, **MTPA-BA**-based PSCs always delivered higher FF and PCE values than devices based on traditional HSC (PTAA or 2PACz). The champion PCE of **MTPA-BA**-based FACs PSCs achieved 21.04%, outstripping the widely used PTAA-based devices as the FF was enhanced from 75.7% to 83.2%. On the other hand, the **MTPA-BA**-based wide-bandgap PSCs can achieve a remarkable FF value of 82.9% (PCE of 19.3%), while the FF value of the 2PACz-based device is barely 76.6%. The detailed photovoltaic parameters of PSCs based on **MTPA-BA** and control HSC are summarized in Supplementary Table 2, and their external quantum efficiency (EQE) and steady-state photocurrent output are plotted in Supplementary Figs 21 and 22, respectively. Figure 4i shows a statistical comparison of the FF value with three PSC devices based on different HSC, demonstrating a universally enhanced FF performance upon implementation of **MTPA-BA**.

### **Stability** enhancement **via HSC** engineering

**Figure 5.**
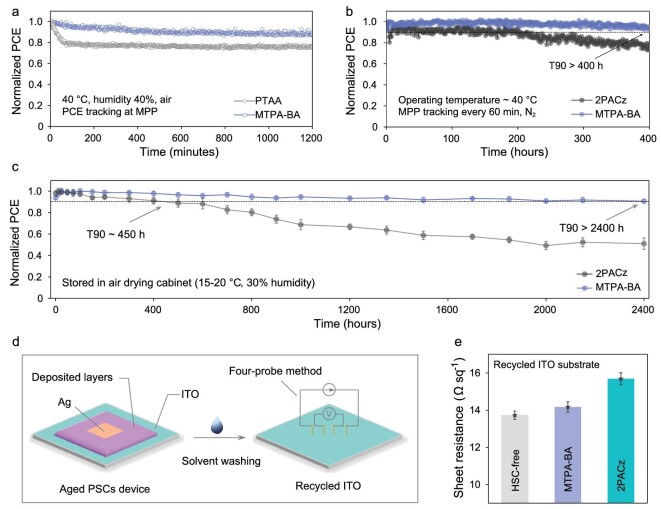
Stability test of PSCs with different HSC. (a) Comparison of operational stability of PTAA- and **MTPA-BA**-based devices by tracking the MPP under simulated AM 1.5-G irradiation (the initial PCE of PTAA- and **MTPA-BA**-based FACs perovskite devices are 18.0% and 20.7%, respectively). (b) MPP tracking of FAMACs-Br_0.05_ PSCs with different HSC under 1 sun equivalent white-light light-emitting diode array illumination (**MTPA-BA**, initial PCE 22.3%, 2PACz, initial PCE 20.8%). (c) Long-term shelf life of **MTPA-BA**- and 2PACz-based FAMACs-Br_0.05_ PSCs (data from three individual devices). (d) Schematic of sheet resistance measurements with recycled ITO from aged device (solvent, DMF/MeOH = 1/9). (e) Sheet resistance changes of recycled ITO substrate coating with different HSC.

The long-term stability of PSCs is the most important aspect to investigate before their real application. The protocol of ISOS-L-1, namely maximum power-point (MPP) tracking under light irradiation, was used to evaluate the operational stability of PSCs with different HSC [44]. Figure 5a shows continuous MPP tracking of unsealed devices under simulated AM 1.5-G sunlight in ambient air. PSCs based on FACs perovskite and **MTPA-BA** HSC can maintain 90% of their initial efficiency after 1200 min. However, PTAA-based devices show an evident decline in the initial 60 min (∼15% PCE loss). The quick initial decrease in PCE could be related to inferior interfacial contact of the perovskite with the HSC. Figure 5b exhibits the continuous MPP tracking of an unsealed device employing FAMACs-Br_0.05_ perovskites under 1 sun equivalent light-emitting diode array illumination in a nitrogen atmosphere. The PCE of the **MTPA-BA**-based devices decreased by merely 10% after continuous light soaking for 400 h, which is also better than the 2PACz-based devices. These results suggest that enhanced interfacial contact can remarkably improve the operational stability of PSC devices.

Furthermore, the storage stability of unsealed PSCs was tracked in an air-drying cabinet according to ISOS-D-1 standard (Supplementary Fig. 23). The **MTPA-BA**-based devices exhibited only a slight PCE drop after 1000 h, proving that boric acid HSC can maintain decent storage stability. Employing T90 (time to 90% of initial PCE) as a standard, the lifespan of **MTPA-BA**- and 2PACz-based devices was compared. As shown in Fig. 5c, the T90 of 2PACz-based PSCs was ∼450 h. In contrast, the **MTPA-BA**-based PSCs achieved T90 of >2400 h, showing a 5-fold increase in shelf life. Considering that light, heat and other stimuli were absent in this test, the improved storage stability can be attributed to the corrosion-less HSC. To prove this conjecture, we recycled ITO from aged devices and tested their sheet resistance by using the four-probe method (Fig. 5d). After storing for 2500 h, the sheet resistance of ITO in the 2PACz-based devices increase by 2.0 Ω sq^−1^ (Fig. 5e), while the ITO in **MTPA-BA**-based devices only increased by ∼0.5 Ω sq^−1^ (versus HSC-free devices). These results verify the suppression of ITO corrosion, consistently with the previous ICP–MS results. Supplementary Fig. 24 compares the performance of PSCs fabricated with recycled ITO substrates. The PCE of the **MTPA-BA**-based device drops by ∼5%, which is smaller than that of 2PACz (∼20%), and the latter showed an inferior MPP stability (Supplementary Table 3). Thus, compared with phosphonic acid-based HSC, the acidity-weakened boric acid-anchoring HSC mitigates the corrosion of ITO, reduces the resistive loss, improves interfacial stability and should be a promising candidate for future commercialization.

## CONCLUSION

In summary, we have demonstrated the feasibility of boric acid-anchoring SAM for inverted PSCs and fully elucidated their working mechanism by studying the adsorption process and interfacial chemical interactions. Impressively, an acidity-weakened boric acid anchor significantly mitigates ITO corrosion and improves interfacial stability. Theoretical calculation reveals that boric acid can easily chemisorb on the ITO surface, with oxygen vacancies facilitating the adsorption progress. By incorporating boric acid onto hole-selective arylamines, we constructed acidity-weakened HSC featuring tunable energy levels, excellent hole selectivity and unique PL properties, especially realizing an improvement in perovskite–substrate contact and buried passivation. Eventually, the optimal boric acid HSC achieved a PCE of close to 23% and a record FF of 85.2% among inverted PSCs, with 5-fold higher shelf life than phosphonic acid HSC-based devices. Moreover, the boric acid HSC was also applicable to MA/Br-free and wide-bandgap PSCs, resulting in a universal enhancement of FF performance. We believe that this study will open new avenues for the development of SAM-based HSC for efficient and durable PSCs.

## MATERIALS AND METHODS

The boric acid compounds used in this work were all purchased from Leyan, Bidepharm and MACKLIN; other materials and solvents can be found in our previous work [17].

Device fabrication can be found in the Supplementary data, mainly referring to the reported literature [45]. Most characterizations such as UV–vis absorption and transmission spectroscopy, XRD, SEM, contact angle, light soaking, cyclic voltammetry measurement, DFT calculation, thermogravimetric and differential scanning calorimetry analysis can be found in our previous work [17]. The sheet resistance of ITO was obtained by using a four-probe tester M-3 (Suzhou Jingge Electronic Co., Ltd). The final results were derived from the average value of 10 times of independent measurements. The indium content of samples was measured by using ICP–MS (NexION 2000-(A-10)). Raman spectra of the sample was recorded by using a 1% power 532-nm laser with ×50 objective lens (Reinshaw invia). Film was observed by using an optical microscope (reflection mode, Nikon LVPOL 100) and captured by using a charge-coupled device camera; the PL image was recorded under an optical microscope with a 365-nm UV flashlight. Confocal PL imaging of the perovskite was captured by using a Leica confocal microscope TCS SPS CFSMP (collect PL signals at 700–790 nm under 514-nm excitation) with a ×60 oil immersion objective lens. UPS was performed by using a SCIENTA R3000 spectrometer under an ultra-high vacuum system (10^−10^ mbar). PL lifetimes were measured by using an Edinburgh Instruments Fluorescence Spectrometer (FLS1000, under 510-nm excitation). The PLQY of the perovskite films was measured under integrating sphere mode (air, Ocean QY) and a 405-nm LED light source was used as excitation. *J–V* curves of PSCs (active area of the device was defined by a metal mask of 0.0625 cm^−2^, scan rate = 50 mV s^−1^, delay time = 100 ms) were measured by using a Keithley 2400 Source meter instrument under standard AM 1.5-G simulated solar irradiation (Zolix Instrument Co., Ltd). Calibration of light intensity was performed with standard silicon cells prior to testing. EQE spectra were measured by using the DSR600 system (Zolix Instruments Co., Ltd).

## Supplementary Material

nwad057_Supplemental_File
