# Supplementary material for: Neglected acidity pitfall: boric acid-anchoring hole-selective contact for perovskite solar cells
Source: Natl Sci Rev. 2023 Mar 3;10(5):nwad057. doi: 10.1093/nsr/nwad057 (PMC10237332; doi:10.1093/nsr/nwad057)
Supplement: nwad057_Supplemental_File [file nwad057_supplemental_file.docx]

Supplementary for

**Neglected Acidity Pitfall: Boric Acid-Anchoring Hole-Selective Contact for Perovskite Solar Cells**

*Huanxin Guo,^1^ Cong Liu,^1^ Honglong Hu**,^1^ Shuo Zhang,^1^ Xiaoyu Ji,^1^ Xiao-Ming Cao,^1^ Zhijun Ning,^2^ Wei-Hong Zhu,^1^ He Tian^1^ and Yongzhen Wu*^,1^*

*^1^* *Key Laboratory for Advanced Materials and Joint International Research Laboratory of Precision Chemistry and Molecular Engineering, Feringa Nobel Prize Scientist Joint Research Center, Institute of Fine Chemicals, Frontiers Science Center for Materiobiology and Dynamic Chemistry, School of Chemistry and Molecular Engineering, East China University of Science and Technology, Shanghai 200237, China E-mail:* *wu.yongzhen@ecust.edu.cn*

*^2^* *School of Physical Science and Technology, Shanghai Tech University, Shanghai 201210, China*

# Supplementary Discussions

# Materials and Device Fabrication

*Materials*. The supplier of lead iodide (PbI_2_), cesium iodide (CsI), methylamine bromide (MABr), methylamine hydrochloride (MACl), formamidine hydroiodic acid (FAI), phenethylamine iodide (PEAI), PCBM, C_60_, BCP, chlorobenzene (CB), ethanol (EtOH) that were used in fabrication of perovskite are all consistent with our previous work [1-3].

*HSC deposition*. The ITO substrates were first washed with a standard procedure and treated with UV ozone (20 min), and then transferred to into a glove box filled with N_2_. Several types of hole-transporting materials were used in this work. We first prepared their solutions with using suitable solvents and concentration (PTAA in chlorobenzene, 2.0 mg mL^-1^ and 2PACz in ethanol, 0.3 mg mL^-1^). MTPA-BA was dissolved in chlorobenzene with concentration range from 0.5-5.0 mg mL^-1^. Moreover, Cz-BA was dissolved in tetrahydrofuran/chlorobenzene = 1/9, MeOTPA-BA and TPA-BA were dissolved in chlorobenzene, and the concentration of the three used in device optimization was 2.5 mg mL^-1^. The HSC solutions were spin coated onto ITO substrate at 4000 rpm for 30 s (acceleration 2000 rpm s^-1^) and heating at 100 ^o^C for 10 min.

*Perovskite deposition*. For FA_0.8_Cs_0.2_PbI_3_ perovskite (1.5 M): 309.6 mg FAI, 116.9 mg CsI and 1037.3 mg PbI_2_ were added into the litter bottle, 1200 µL DMF and 300 µL DMSO were dropped into bottle. Stirring at 50 ^o^C overnight. Preparation process: 6000 rpm for 30 s, 400 µL CB is punched in 8-10 s before the ending, and then annealed at 100 ^o^C (10 min). For Cs_0.05_(FA_0.95_MA_0.05_)_0.95_Pb(I_0.95_Br_0.05_)_3_ perovskite (1.5 M): Prepare 1.5 M solutions of FAPbI_3_ and MAPbBr_3_, respectively (put DMF/DMSO solution of PbI_2_ or PbBr_2_ into the corresponding cationic salts), in which PbI_2_ and PbBr_2_ are in excess of 9%, moreover, configure CsI (1.65 M, DMSO) and MACl (3.5 M, DMSO) separately. Before preparing perovskite film, the four components were successively mixed with a volume ratio of 1200, 62, 67, and 70 µL, respectively. Shaken up and filtered for use. Preparation process: 5000 rpm for 40 s (accelerated completion within five seconds), 250 µL CB is punched in 5-10 s before the ending, and then annealed at 100 ^o^C (60 min). For Cs_0.05_(FA_0.77_MA_0.23_)_0.95_Pb(I_0.77_Br_0.23_)_3_ perovskite (1.5 M): The previous process is the same as the above mentioned, but the volume ratio of different components is different. Here 1200 µL FAPbI_3_, 360 µL MAPbBr_3_ and 51 µL CsI are added to a bottle and shaken before use. Preparation process: 6000 rpm for 50 s (acceleration 1200 rpm s^-1^), 400 µL CB/anisole is punched in 30-35 s after beginning of spin coating, and then annealed at 100 ^o^C (20 min). The anti-solvent needs to be dripped within 1 s to ensure the overall brightness of film.

*Device completion*. After finishing the perovskite preparation, a series of functional layers were deposited to complete the solar cell devices. A passivation layer (PEAI, 1.25 mg mL^-1^ in isopropyl alcohol) and an electron-transporting layer (PCBM, 2.5 mg mL^-1^ in chlorobenzene) were deposited on top of perovskite layer by spin-coating at 6000 rpm for 30 s. Then C_60_ (25 nm), BCP (7 nm) and silver (100 nm) layers were sequential deposited by thermal evaporation in 10^-5^ Pa.

# Calculation Details

All the calculations were performed within periodic plane wave framework and using GGA-PBE functional implemented in VASP package. The projector augmented wave (PAW) pseudopotential was used to model core electrons and the plane wave basis-set was expanded to a converged cut-off energy of 450 eV. Van der Waals interaction was considered by empirical D3-BJ method of Grimme et al [4].

The ITO was modeled by the bulk In_2_O_3_ with doped Sn. For the most stable (111) surface, it is featured with the trilayer structure with a 6-fold coordinated In at each layer. The ITO (111) surface was simulated by a 4-trilayers slab with the *p* (1×1) supercell. The slab separation was provided normal to the surface by inclusion of a 20 Å vacuum region. A mesh of 2×2×1 Monkhorst-Pack k-points was used for the Brillouin-zone integration. The bottom two trilayers were fixed while the other trilayers and adsorbates were allowed to relax during the geometry optimization. The geometry convergence criterion was set as 0.05 eV/Å for the maximal component of force.

The most frequently exposed surfaces of several oxides were used to simulate the oxygen vacancy formation, respectively corresponding to Al_2_O_3_ (001) [5], quartz-SiO_2_ (001) [6], anatase-TiO_2_ (101) [7], and SnO_2_ (110) [8]. All the surfaces were simulated by a 4-layers slab with the *p* (2×2) supercell. The slab separation was provided normal to the surface by inclusion of a 20 Å. The bottom two layers were fixed while the other layers were allowed to relax during the geometry optimization. The geometry convergence criterion was set as 0.05 eV/Å for the maximal component of force.

*COHP*: The integration of the contribution of the energy bands up to the Fermi level based on the projected crystal orbital Hamilton population (IpCOHP) analysis indicates the bonding strength between the functional groups and surface. To be analogous to the Crystal Orbital Overlap Population (COOP), -COHP is plotted instead of COHP hereinafter. The more positive the -IpCOHP is, the stronger bonding exists. The greatest -IpCOHP value of O (MTPA-BA)-In (ITO) indicates the strongest chemical bonding between the MTPA-BA and ITO (111) surface.

*Free Energy Calculations*: The Gibbs free energy change (Δ*G*) were calculated as follows:

$\Delta G=\Delta E+\Delta ZPE-\Delta TS$ (1)

where ZPE is the change of zero-point vibrational enthalpy, and TS is the entropy.

*Oxygen Vacancy Formation Energies Calculations*: The formation energies of oxygen vacancy (*E*_Ov_) were calculated as follows:

$E_{O_{V}}=E_{O_{V}-\mathrm{sur}}-E_{\mathrm{sur}}+\frac{1}{2}E_{O_{2}}$ (2)

where *E*_sur_ is the total energy of the bare surface for oxide, and *E*_Ov-sur_ is the total energy of surface with oxygen vacancy, and *E*_O2_ is the total energy of an isolated oxygen molecule.

# Expressions for Solar Cell FF

$\mathrm{FF}_{\max}=\frac{v_{oc}-ln(v_{oc}+0.72)}{v_{oc}+1}$, with $v_{oc}=\frac{qV_{OC}}{nk_{B}T}$ (3)

$\mathrm{FF}_{s}=\mathrm{FF}_{\max}\left( 1-1.1r_{s} \right)+\frac{{r_{s}}^{2}}{5.4}$, with $r_{s}=\frac{J_{SC}R_{s}}{V_{OC}}$ (4)

$\mathrm{FF}=\mathrm{FF}_{s}\left( 1-\frac{v_{oc}+0.72}{v_{oc}}\frac{\mathrm{FF}_{s}}{r_{\mathrm{sh}}} \right)$, with $r_{\mathrm{sh}}=\frac{J_{SC}R_{\mathrm{sh}}}{V_{OC}}$ (5)

where n represents ideality factor (quantized from the slope of the *V*_OC_ versus light intensity), an upper *V*_OC_ limit of 1.28 V was assumed to calculate the radiative FF limit. *r*_s_ and *r*_sh_ are the normalized resistances. FF_max_ represents a potential FF of PSCs device without considering charge transport losses.

# Supplementary Figures

**DFT Calculation for Boric Acid HSC**





**Supplementary** **Figure 1**. DFT calculation (the basis set is b3lyp/6-311g*) of boric acid molecules with different arylamine-based hole-transporting moieties (carbazole based Cz-BA and triphenylamine based TPA-BA, MTPA-BA, MeOTPA-BA). a) Molecular structure of boric acid HSC. b) HOMO orbital distributions, c) LUMO orbital distributions, d) Comparison of calculated HOMO values (vs. vacuum) of different boric acid molecule. It can be found that the HOMO was up-shifted with increasing the electron-donating property of the arylamine.

# PL Intensity and Imaging of Boric Acid HSC





**Supplementary** **Figure 2**. a) Photograph of solutions of MTPA-BA (CB, 2.5 mg mL^-1^), 2PACz (EtOH, 2.5 mg mL^-1^), MTPA (CB, 2.5 mg mL^-1^) taken under bright field (left) and in the dark with UV light (365 nm) irradiation, respectively. We note that the MTPA-BA show bright blue fluorescence, while the 2PACz is not emissive. The PL of MTPA is moderate, indicating that the incorporation of boric acid group enhances the PL emission. b-d) The PL images (up, excited by 365 nm UV light) and normal optical images (bottom) of ITO/MTPA-BA films fabricated with different MTPA-BA concentration (5, 2.5, 0.5 mg mL^-1^, scale bar 200 µm, red circles marked as distinct film defects like MTPA-BA particles). The bright emission can help us to roughly judge the uniformity of HSC on ITO substrate.

# Electrochemically Stabilized and Corrosion-Reduced Boric Acid HSC





**Supplementary** **Figure 3**. a) CV results of MTPA-BA in dichloromethane, with ferrocene as the internal standard. b) Excellent electrochemical stability of MTPA-BA under 10 consecutive CV scans.





**Supplementary Figure 4.** a) Thermogravimetric analysis (TGA) and b) differential scanning calorimetry (DSC) curves of MTPA-BA (10 ^o^C min^-1^, N_2_). c) UV-vis absorption evolution of MTPA-BA dilute solution (dichloromethane, 5 × 10^-5^ M) under one sun continuous illumination for 12 hours. The solution remains colorless and similar absorption after 12 h light soaking, proving the decent photostability of MTPA-BA.





**Supplementary** **Figure 5**. a) The indium content of aged solution (10 mg mL^-1^, stirring at 50 ^o^C for 48 h) detected by ICP-MS, N-ethylcarbazole denote as N-EtCz, 4,4'-dimethyl-triphenylamine denote as MTPA. b) Chemical structure of the four compounds.

# Stable B-O-M Covalent Linkage Between Boric Acid and ITO Substrate





**Supplementary** **Figure 6**. The B 1s signal in XPS results of MTPA-BA film fabricated on different substrates a) quartz glass, b) Si substrate.





**Supplementary** **Figure 7**. The B 1s signal in XPS results of MTPA-BA film fabricated on ITO substrates. During the fabrication of this sample, the MTPA-BA solution was placed on ITO for 30 s before spin-coating. Compared to direct spin-coating, the ratio of B-O-M signal were doubled from 25.0% to 50.0%, suggesting quick and efficient SAM formation.





**Supplementary** **Figure 8**. XPS results of ITO/MTPA-BA film with or without chlorobenzene washing: a) N 1s spectra, b) B 1s spectra, control is Fig. 2a. After solvent washing, the sample still retain the unique N 1s and B 1s signals, indicating the stable anchoring of boric acid HSC on ITO substrate.





**Supplementary** **Figure 9**. The In 3d signal in XPS result of ITO and ITO/MTPA-BA samples. Comparing with the blank ITO, the In 3d signal of ITO/**MTPA-BA** sample exhibited a negative energy shift (~ 0.4 eV, 0.2 eV for washed sample) regardless of washing, indicating the strong chemical binding between boric acid and ITO substrate.

# Crystal Planes used in Calculation and Oxygen Vacancy Results





**Supplementary** **Figure 10**. a) Top view (left) and front view (right) of the optimized ITO (111) surface and b) oxygen vacancy (O_V_) site of ITO. (The red, brown and dark green balls represent O, In and Sn atoms, respectively).





**Supplementary** **Figure 11**. Top (the left column) and side (the right column) views of a) Al_2_O_3_ (001), b) quartz-SiO_2_ (001), c) anatase-TiO_2_ (101), d) SnO_2_ (110) crystalline planes, the red balls represent O atom, and the other colored balls represent metal ions.





**Supplementary** **Figure 12**. The calculated formation energies of oxygen vacancy in common oxides.

# Boric Acid Anchoring HSC Enables High-Quality Perovskite Deposition





**Supplementary** **Figure 13**. Time-dependent contact angles of perovskite inks on three different HSC. Comparing with PTAA and 2PACz, the MTPA-BA coated ITO substrate showed enhanced affinity for perovskite precursor solutions, as revealed by the quick solution spread. This is beneficial to achieve an intimate contact between perovskite and substrate.





**Supplementary** **Figure 14**. PLQY of triplet-cation perovskite film deposited on MTPA-BA (405 nm excitation, with quartz glass substrate as a control). The different concentration (0.5, 2.5, 5.0 mg mL^-1^) of MTPA-BA solutions used for the HSC deposition played a important role on the PLQY of perovskite films.





**Supplementary** **Figure 15**. SEM images of FAMACs-Br_0.05_ perovskite films deposited on MTPA-BA HSC layer fabricated with different concentration (scale bar 1 µm).





**Supplementary** **Figure 16**. XRD of FAMACs-Br_0.05_ perovskite film fabricated on MTPA-BA HSC layer fabricated with different concentration (0.5, 2.5, 5 mg mL^-1^).





**Supplementary** **Figure 17**. a) Cross-sectional SEM image of inverted PSCs employing FAMACs-Br_0.05_ and MTPA-BA as absorber and HSC, respectively. b) Configuration schematic of inverted PSCs.

# Photovoltaic Performance of Boric Acid Anchoring HSC





**Supplementary** **Figure 18**. Statistical distribution of photovoltaic parameter for triplet-cation FAMACs-Br_0.05_ based PSCs with different boric acid HSC (Cz-BA, TPA-BA, MTPA-BA, MeOTPA-BA).





**Supplementary** **Figure 19**. Statistical distribution of photovoltaic parameter for FACs perovskite based PSCs with different HSC (PTAA, MTPA-BA, MTPA and HSC-free substrate).





**Supplementary** **Figure 20**. Dark *J-V* curves of MTPA-BA and 2PACz based device. The perovskite in this devices is triplet-cation FAMACs-Br_0.05_.





**Supplementary** **Figure 21**. EQE results of PSCs based on different perovskite compositions.





**Supplementary** **Figure 22**. Stabilized photocurrent output of different PSCs under a bias voltage near the MPP.





**Supplementary** **Figure 23**. Storage stability evolution of three PSCs based on MTPA-BA.





**Supplementary** **Figure 24**. a) *J-V* curves, b) steady-state photocurrent output and c) photovoltaic parameter distributions of PSC devices fabricated on fresh and recycled ITO substrates. BA, PA are boric acid and phosphoric acid, here represent MTPA-BA and 2PACz, respectively. The decrease of device performance is mainly concentrated in *J*_SC_ and FF, which is related to the irreversible optical damage and increased sheet resistance of recycled ITO (2PACz based device decreases ~ 20%, MTPA-BA based device drops ~ 5%).

# Supplementary Table

**Supplementary** **Table 1**. Series resistance and shunt resistance data of the champion devices of FAMACs-Br_0.05_ perovskites with different HSC (reverse scan) for FF calculation.

| HSC | *R*_s_ | *R*_sh_ | FF_max_ | FF_s_ | FF_cal_ |
| --- | --- | --- | --- | --- | --- |
| MTPA-BA-R | 1.19 | 7515.28 | 87.0% | 84.7% | 84.2% |
| 2PACz-R | 3.37 | 5395.82 | 85.8% | 79.4% | 78.8% |

**Supplementary** **Table 2**. Summary of device parameters of different perovskite and HSC (reverse and forward scan, scan rate 50 mV s^-1^).

| Perovskite | HSC | *V*_OC_ (V) | *J*_SC_ (mA cm^-2^) | FF (%) | PCE (%) |
| --- | --- | --- | --- | --- | --- |
| Cs_0.05_(FA_0.95_MA_0.05_)_0.95_Pb(I_0.95_Br_0.05_)_3_ | MTPA-BA-R | 1.14 | 23.24 | 85.2 | 22.62 |
|  | MTPA-BA-F | 1.14 | 23.29 | 84.1 | 22.35 |
|  | Control-R | 1.14 | 23.06 | 80.1 | 21.05 |
|  | Control-F | 1.14 | 23.07 | 78.9 | 20.70 |
| FA_0.8_Cs_0.2_PbI_3_ | MTPA-BA-R | 1.10 | 23.00 | 83.2 | 21.04 |
|  | MTPA-BA-F | 1.09 | 23.04 | 82.3 | 20.66 |
|  | Control-R | 1.10 | 22.78 | 75.7 | 18.96 |
|  | Control-F | 1.09 | 22.63 | 74.4 | 18.35 |
| Cs_0.05_(FA_0.77_MA_0.23_)_0.95_Pb(I_0.77_Br_0.23_)_3_ | MTPA-BA-R | 1.16 | 20.09 | 82.9 | 19.28 |
|  | MTPA-BA-F | 1.15 | 20.20 | 80.7 | 18.74 |
|  | Control-R | 1.17 | 19.59 | 76.6 | 17.56 |
|  | Control-F | 1.17 | 19.88 | 74.0 | 17.21 |

**Supplementary** **Table 3**. Device performance parameters (reverse scan) of two HSC based on fresh and recycled ITO substrates.

| Perovskite | ITO | HSC | *V*_OC_ (V) | *J*_SC_ (mA cm^-2^) | FF (%) | PCE (%) |
| --- | --- | --- | --- | --- | --- | --- |
| Cs_0.05_(FA_0.95_MA_0.05_)_0.95_Pb(I_0.95_Br_0.05_)_3_ | Fresh | MTPA-BA | 1.133 | 23.07 | 84.39 | 22.05 |
|  | Recycled | MTPA-BA | 1.127 | 22.47 | 81.53 | 20.65 |
|  | Fresh | 2PACz | 1.129 | 22.90 | 79.20 | 20.48 |
|  | Recycled | 2PACz | 1.126 | 21.01 | 68.30 | 16.17 |

# Supplementary Reference

1. Li E, Liu C and Lin H *et al.* Bonding strength regulates anchoring‐based self‐assembly monolayers for efficient and stable perovskite solar cells. *Adv Funct Mater* 2021; **31**: 2103847.
2. Guo H, Zhang H and Shen C *et al.* A coplanar π‐extended quinoxaline based hole-transporting material enabling over 21% efficiency for dopant‐free perovskite solar cells. *Angew Chem Int Ed* 2021; **60**: 2674–79.
3. Guo H, Zhang H and Liu S *et al.* Efficient and stable methylammonium-free tin-lead perovskite solar cells with hexaazatrinaphthylene-based hole-transporting materials. *ACS Appl Mater Interfaces* 2022; **14**: 6852–58.
4. Grimme S, Ehrlich S and Goerigk L. Effect of the damping function in dispersion corrected density functional theory. *J Comput Chem* 2011; **32**: 1456–65.
5. Tamijani AA, Bjorklund JL and Augustine LJ *et al.* Density functional theory and thermodynamics modeling of inner-sphere oxyanion adsorption on the hydroxylated α-Al_2_O_3_ (001) surface. *Langmuir* 2020; **36**: 13166–80.
6. Goumans TPM, Wander A and Brown WA *et al.* Structure and stability of the (001) α-quartz surface. *Phys Chem Chem Phys* 2007; **9**: 2146–52.
7. Li Y-F and Selloni A. Pathway of photocatalytic oxygen evolution on aqueous TiO_2_ anatase and insights into the diﬀerent activities of anatase and rutile. *ACS Catal* 2016; **6**: 4769–74.
8. Wang X, Qin H and Chen Y *et al.* Sensing mechanism of SnO_2_ (110) surface to CO: density functional theory calculations. *J Phys Chem C* 2014; **118**: 28548–61.
